# Supplementary material for: Modeling glioblastoma heterogeneity as a dynamic network of cell states
Source: Mol Syst Biol. 2021 Sep 16;17(9):e10105. doi: 10.15252/msb.202010105 (PMC8444284; doi:10.15252/msb.202010105)
Supplement: Supplementary file 5 — Source Data for Figure 3 [file MSB-17-e10105-s001.zip › Figure3A_sourcedata/GSEA_3065/hallmarks_state4.GseaPreranked.1623416296068/HALLMARK_EPITHELIAL_MESENCHYMAL_TRANSITION.html]

Details for gene set HALLMARK\_EPITHELIAL\_MESENCHYMAL\_TRANSITION[GSEA]

|  || Dataset | state4 |
| Phenotype | NoPhenotypeAvailable |
| Upregulated in class | na\_neg |
| GeneSet | HALLMARK\_EPITHELIAL\_MESENCHYMAL\_TRANSITION |
| Enrichment Score (ES) | -0.49216864 |
| Normalized Enrichment Score (NES) | -1.7580767 |
| Nominal p-value | 0.0 |
| FDR q-value | 0.0045889434 |
| FWER p-Value | 0.04 |
Table: GSEA Results Summary

  

Fig 1: Enrichment plot: HALLMARK\_EPITHELIAL\_MESENCHYMAL\_TRANSITION      
 Profile of the Running ES Score & Positions of GeneSet Members on the Rank Ordered List

  

| PROBE | GENE SYMBOL | GENE\_TITLE | RANK IN GENE LIST | RANK METRIC SCORE | RUNNING ES | CORE ENRICHMENT || 1 | SAT1 |  |  | 4 | 0.669 | 0.0448 | No |
| 2 | PFN2 |  |  | 46 | 0.303 | 0.0587 | No |
| 3 | ID2 |  |  | 56 | 0.292 | 0.0771 | No |
| 4 | APLP1 |  |  | 102 | 0.246 | 0.0866 | No |
| 5 | PTX3 |  |  | 238 | 0.188 | 0.0775 | No |
| 6 | DPYSL3 |  |  | 298 | 0.174 | 0.0797 | No |
| 7 | PRRX1 |  |  | 383 | 0.157 | 0.0768 | No |
| 8 | NNMT |  |  | 448 | 0.144 | 0.0762 | No |
| 9 | COL1A2 |  |  | 466 | 0.142 | 0.0830 | No |
| 10 | SPARC |  |  | 539 | 0.132 | 0.0803 | No |
| 11 | EFEMP2 |  |  | 727 | 0.111 | 0.0575 | No |
| 12 | PMP22 |  |  | 765 | 0.107 | 0.0587 | No |
| 13 | MMP2 |  |  | 813 | 0.103 | 0.0581 | No |
| 14 | COL11A1 |  |  | 905 | 0.095 | 0.0498 | No |
| 15 | SLC6A8 |  |  | 926 | 0.093 | 0.0529 | No |
| 16 | HTRA1 |  |  | 1008 | 0.088 | 0.0457 | No |
| 17 | WIPF1 |  |  | 1111 | 0.082 | 0.0347 | No |
| 18 | ITGB5 |  |  | 1183 | 0.077 | 0.0284 | No |
| 19 | CAP2 |  |  | 1276 | 0.072 | 0.0184 | No |
| 20 | CDH11 |  |  | 1280 | 0.072 | 0.0227 | No |
| 21 | VIM |  |  | 1350 | 0.069 | 0.0162 | No |
| 22 | FSTL1 |  |  | 1367 | 0.068 | 0.0182 | No |
| 23 | SNTB1 |  |  | 1401 | 0.066 | 0.0174 | No |
| 24 | FERMT2 |  |  | 1426 | 0.065 | 0.0179 | No |
| 25 | PDLIM4 |  |  | 1446 | 0.064 | 0.0192 | No |
| 26 | MSX1 |  |  | 2143 | 0.043 | -0.0908 | No |
| 27 | MATN2 |  |  | 2545 | 0.034 | -0.1536 | No |
| 28 | COL5A2 |  |  | 2672 | 0.031 | -0.1719 | No |
| 29 | CALD1 |  |  | 2682 | 0.031 | -0.1712 | No |
| 30 | CD44 |  |  | 2687 | 0.031 | -0.1698 | No |
| 31 | RHOB |  |  | 2911 | 0.026 | -0.2042 | No |
| 32 | LRP1 |  |  | 2940 | 0.026 | -0.2069 | No |
| 33 | FGF2 |  |  | 3038 | 0.024 | -0.2210 | No |
| 34 | FLNA |  |  | 3061 | 0.024 | -0.2230 | No |
| 35 | CD59 |  |  | 3163 | 0.022 | -0.2379 | No |
| 36 | TGFB1 |  |  | 3283 | 0.019 | -0.2559 | No |
| 37 | LGALS1 |  |  | 3527 | 0.011 | -0.2946 | No |
| 38 | GAS1 |  |  | 3553 | 0.011 | -0.2979 | No |
| 39 | VEGFA |  |  | 3787 | 0.001 | -0.3356 | No |
| 40 | CADM1 |  |  | 3798 | 0.001 | -0.3372 | No |
| 41 | NOTCH2 |  |  | 3944 | -0.006 | -0.3603 | No |
| 42 | BMP1 |  |  | 3952 | -0.007 | -0.3610 | No |
| 43 | JUN |  |  | 3979 | -0.008 | -0.3647 | No |
| 44 | COL5A1 |  |  | 3991 | -0.009 | -0.3658 | No |
| 45 | PCOLCE |  |  | 4002 | -0.010 | -0.3668 | No |
| 46 | FUCA1 |  |  | 4070 | -0.013 | -0.3767 | No |
| 47 | COPA |  |  | 4071 | -0.014 | -0.3758 | No |
| 48 | GJA1 |  |  | 4123 | -0.017 | -0.3829 | No |
| 49 | FN1 |  |  | 4127 | -0.017 | -0.3822 | No |
| 50 | VCAN |  |  | 4158 | -0.019 | -0.3858 | No |
| 51 | COL16A1 |  |  | 4338 | -0.031 | -0.4127 | No |
| 52 | COL4A1 |  |  | 4362 | -0.033 | -0.4142 | No |
| 53 | NTM |  |  | 4381 | -0.034 | -0.4148 | No |
| 54 | MYLK |  |  | 4481 | -0.043 | -0.4280 | No |
| 55 | ECM1 |  |  | 4645 | -0.055 | -0.4507 | No |
| 56 | MEST |  |  | 4739 | -0.063 | -0.4615 | No |
| 57 | THBS1 |  |  | 4747 | -0.063 | -0.4583 | No |
| 58 | PLOD2 |  |  | 4764 | -0.065 | -0.4565 | No |
| 59 | CDH2 |  |  | 4839 | -0.071 | -0.4637 | No |
| 60 | LOXL1 |  |  | 4852 | -0.072 | -0.4607 | No |
| 61 | ADAM12 |  |  | 4896 | -0.075 | -0.4626 | No |
| 62 | CXCL12 |  |  | 5079 | -0.089 | -0.4861 | Yes |
| 63 | PLOD3 |  |  | 5090 | -0.091 | -0.4815 | Yes |
| 64 | GLIPR1 |  |  | 5091 | -0.091 | -0.4753 | Yes |
| 65 | COLGALT1 |  |  | 5095 | -0.091 | -0.4696 | Yes |
| 66 | TPM1 |  |  | 5108 | -0.092 | -0.4653 | Yes |
| 67 | SERPINH1 |  |  | 5124 | -0.094 | -0.4614 | Yes |
| 68 | COL4A2 |  |  | 5156 | -0.098 | -0.4598 | Yes |
| 69 | PLAUR |  |  | 5171 | -0.099 | -0.4553 | Yes |
| 70 | TAGLN |  |  | 5209 | -0.102 | -0.4544 | Yes |
| 71 | DST |  |  | 5242 | -0.105 | -0.4525 | Yes |
| 72 | GADD45A |  |  | 5297 | -0.109 | -0.4538 | Yes |
| 73 | ITGAV |  |  | 5309 | -0.110 | -0.4481 | Yes |
| 74 | THY1 |  |  | 5325 | -0.111 | -0.4430 | Yes |
| 75 | SGCB |  |  | 5334 | -0.112 | -0.4367 | Yes |
| 76 | GPC1 |  |  | 5348 | -0.113 | -0.4311 | Yes |
| 77 | TNC |  |  | 5359 | -0.114 | -0.4250 | Yes |
| 78 | SDC4 |  |  | 5709 | -0.161 | -0.4706 | Yes |
| 79 | IGFBP2 |  |  | 5777 | -0.179 | -0.4693 | Yes |
| 80 | LAMC1 |  |  | 5795 | -0.182 | -0.4598 | Yes |
| 81 | SERPINE1 |  |  | 5812 | -0.186 | -0.4497 | Yes |
| 82 | TPM4 |  |  | 5876 | -0.200 | -0.4463 | Yes |
| 83 | ITGB1 |  |  | 5877 | -0.201 | -0.4327 | Yes |
| 84 | SERPINE2 |  |  | 5913 | -0.210 | -0.4241 | Yes |
| 85 | SDC1 |  |  | 5998 | -0.237 | -0.4217 | Yes |
| 86 | PMEPA1 |  |  | 6009 | -0.241 | -0.4069 | Yes |
| 87 | PPIB |  |  | 6033 | -0.256 | -0.3933 | Yes |
| 88 | CALU |  |  | 6049 | -0.268 | -0.3775 | Yes |
| 89 | NT5E |  |  | 6064 | -0.277 | -0.3610 | Yes |
| 90 | EMP3 |  |  | 6085 | -0.287 | -0.3447 | Yes |
| 91 | PTHLH |  |  | 6141 | -0.330 | -0.3312 | Yes |
| 92 | IGFBP3 |  |  | 6184 | -0.397 | -0.3111 | Yes |
| 93 | TIMP1 |  |  | 6191 | -0.409 | -0.2843 | Yes |
| 94 | DKK1 |  |  | 6208 | -0.466 | -0.2552 | Yes |
| 95 | TFPI2 |  |  | 6230 | -0.518 | -0.2234 | Yes |
| 96 | SPP1 |  |  | 6231 | -0.520 | -0.1881 | Yes |
| 97 | TNFRSF12A |  |  | 6251 | -0.658 | -0.1464 | Yes |
| 98 | MCM7 |  |  | 6252 | -0.665 | -0.1012 | Yes |
| 99 | POSTN |  |  | 6256 | -0.753 | -0.0505 | Yes |
| 100 | SCG2 |  |  | 6257 | -0.758 | 0.0010 | Yes |
Table: GSEA details [plain text format]

  

Fig 2: HALLMARK\_EPITHELIAL\_MESENCHYMAL\_TRANSITION: Random ES distribution      
 Gene set null distribution of ES for **HALLMARK\_EPITHELIAL\_MESENCHYMAL\_TRANSITION**
